# Ginger Supplementation Improves Performance and Attenuates Perceived Exertion and Neuromuscular Fatigue in CrossFit^®^ Athletes

**DOI:** 10.3390/jfmk11030301

**Published:** 2026-07-29

**Authors:** Erick Barbalho Lage, Naiara Ribeiro Almeida, Sebastian Sepúlveda González, Felipe Inostroza-Rios, José Raimundo Fernandes, Michele Andrade de Brito, Diego Valenzuela Pérez, Dany Alexis Sobarzo Soto, Esteban Aedo-Muñoz, Felipe J. Aidar, Ciro José Brito

**Affiliations:** 1 Department of Physical Education, Federal University of Juiz de Fora, Campus Governador Valadares, Governador Valadares 35010-180, Minas Gerais, Brazil; erick.lage@estudante.ufjf.br (E.B.L.); naiara.ribeiro@ufjf.br (N.R.A.); ssepulvedag17@gmail.com (S.S.G.); felipe.inostroza.311@gmail.com (F.I.-R.); 2 Vale do Rio Doce University, Governador Valadares 35010-180, Minas Gerais, Brazil; mundegv@hotmail.com (J.R.F.); michele.psi1980@gmail.com (M.A.d.B.); 3 Facultad de Salud y Ciencias Sociales, Universidad de las Américas, Santiago 8370040, Chile; dvalenzuelap@udla.cl; 4 Facultad de Salud, Magister en Ciencias de la Actividad Física y Deportes Aplicadas al Entrenamiento Rehabilitación y Reintegro Deportivo, Escuela de Kinesiología, Universidad Santo Tomás, Puerto Montt 5480000, Chile; danysobarzo@santotomas.cl; 5 Escuela de Educación Física, Deportes y Recreación, Universidad Metropolitana de Ciencias de la Educación, Santiago 7760197, Chile; esteban.aedo@umce.cl; 6 Graduate Program of Physiological Science, Graduate Program of Movement Sciences, Federal University of Sergipe, São Cristóvão 49100-000, Sergipe, Brazil; fjaidar@gmail.com; 7 Graduate Program of Physical Education, Federal University of Viçosa, Campus Florestal, Florestal 35690-000, Minas Gerais, Brazil

**Keywords:** CrossFit^®^, supplementation, ginger, physical performance, high-intensity exercise

## Abstract

**Objective:** To investigate the effects of short-term ginger supplementation on physical performance, perceived exertion, and neuromuscular fatigue in CrossFit^®^ practitioners. **Methods:** Thirty-two CrossFit^®^ practitioners were randomly assigned to a Ginger group (n = 17) or a Placebo group (n = 15) in a randomized, double-blind, placebo-controlled trial. Participants consumed 2 g·day^−1^ of ginger or placebo for 9 days. Performance was assessed using a 10 min As Many Repetitions As Possible (AMRAP) test. Ratings of perceived exertion (RPE) and countermovement jump (CMJ) variables were evaluated before and after exercise. **Results:** Ginger supplementation significantly improved AMRAP performance compared with placebo (241.6 ± 18.2 vs. 225.9 ± 17.5 repetitions; F_1,29_ = 5.95, *p* = 0.021, ηp^2^ = 0.17). Participants in the Ginger group also reported lower RPE values (F_1,29_ = 24.11, *p* ≤ 0.001, ηp^2^ = 0.45). Regarding neuromuscular fatigue, ginger attenuated the decline in CMJ peak force following exercise (F_1,29_ = 6.62, *p* = 0.015, ηp^2^ = 0.18). No significant between-group differences were found for peak power and center of mass displacement. **Conclusions:** Short-term ginger supplementation improved AMRAP performance, reduced perceived exertion, and attenuated the decline in peak force following exercise in CrossFit^®^ practitioners. These findings suggest that ginger supplementation may have potential as a natural ergogenic strategy for high-intensity functional exercise; however, further studies with larger samples are needed to confirm these effects.

## 1. Introduction

CrossFit^®^ is a training methodology and competitive sport characterized by the performance of constantly varied functional movements executed at high intensity, integrating exercises derived from Olympic weightlifting, gymnastics, and cyclical activities such as running and rowing [1,2]. Its primary objective is to simultaneously develop multiple physical capacities, including cardiorespiratory endurance, strength, power, speed, coordination, and agility, distinguishing it from traditional training approaches that typically emphasize specific fitness components in isolation [2,3]. Over the past decades, CrossFit^®^ has evolved into an established competitive sport, largely driven by the development of the CrossFit^®^ Games and its international qualification system [1]. Consequently, athletes are routinely exposed to substantial physiological demands, requiring the maintenance of high levels of performance under conditions of accumulated metabolic and neuromuscular fatigue [1,3,4].

Given these demands, the ability to monitor and mitigate neuromuscular fatigue has become a critical aspect of both performance optimization and athlete health. Acute reductions in explosive power and force-generating capacity may compromise athletic performance and increase the risk of injury [4,5]. In this regard, the countermovement jump (CMJ) has emerged as an important and practical tool for assessing neuromuscular status, owing to its sensitivity in detecting acute alterations in lower-body power production and movement efficiency following high-intensity exercise [4,6]. Considering the high physiological stress associated with CrossFit^®^, strategies aimed at attenuating fatigue and enhancing recovery have received increasing attention. In this context, ergogenic aids have become widely used among practitioners to improve performance, reduce fatigue, and accelerate recovery [7,8].

Although supplements such as caffeine and creatine have been extensively investigated in sports settings, growing interest has been directed toward natural compounds with ergogenic potential [7]. Among these, ginger (*Zingiber officinale*) has attracted considerable attention due to its high concentration of bioactive compounds, including gingerols and shogaols, which possess anti-inflammatory, antioxidant, and analgesic properties [9,10]. Evidence suggests that ginger supplementation may reduce delayed-onset muscle soreness, attenuate inflammatory responses, and decrease exercise-induced oxidative stress, factors that may contribute to improved recovery and the maintenance of physical performance [10,11,12].

Despite these promising findings, the evidence regarding the effects of ginger on exercise performance remains inconclusive. Such inconsistencies may be attributable to differences in supplementation protocols, dosage, intervention duration, and exercise modalities across studies [9,13,14]. Moreover, the existing literature has predominantly focused on traditional aerobic or resistance exercise, whereas investigations involving high-intensity, functional, and multimodal activities such as CrossFit^®^ remain limited [15,16]. Importantly, the potential role of ginger in preserving neuromuscular function and attenuating acute fatigue, as assessed through objective measures such as the CMJ, has not been thoroughly explored in populations engaged in high-intensity functional training [13,17,18]. Although previous studies have used supplementation periods ranging from acute administration to several weeks, short-term protocols lasting approximately 5–11 days have consistently demonstrated beneficial effects on exercise-induced muscle soreness, inflammatory responses, and recovery following strenuous exercise [14,16,19]. Based on this evidence, a 9-day supplementation period was selected as a pragmatic intervention expected to provide sufficient exposure to ginger bioactive compounds while maintaining high participant adherence.

Therefore, further investigation into the ergogenic potential of ginger using sport-specific protocols is warranted. In addition to its anti-inflammatory and antioxidant properties, ginger may contribute to the preservation of neuromuscular function by reducing exercise-induced nociceptor sensitization, limiting secondary muscle damage, and attenuating oxidative stress, mechanisms that may help maintain muscle contractile function during repeated high-intensity efforts [10,11,16]. The AMRAP (As Many Repetitions As Possible) format, commonly employed in both CrossFit^®^ training and competition, provides an ecologically valid model for evaluating performance under conditions of high physiological demand [20]. Accordingly, the present study aimed to investigate the effects of nine days of ginger supplementation on physical performance, physiological responses, and neuromuscular status during a 10 min AMRAP test in CrossFit^®^ practitioners. We hypothesized that ginger would increase the total number of repetitions completed, accompanied by lower ratings of perceived exertion and superior maintenance of neuromuscular performance (as measured by the CMJ) compared with placebo.

## 2. Materials and Methods

### 2.1. Experimental Approach

**Figure 1:**
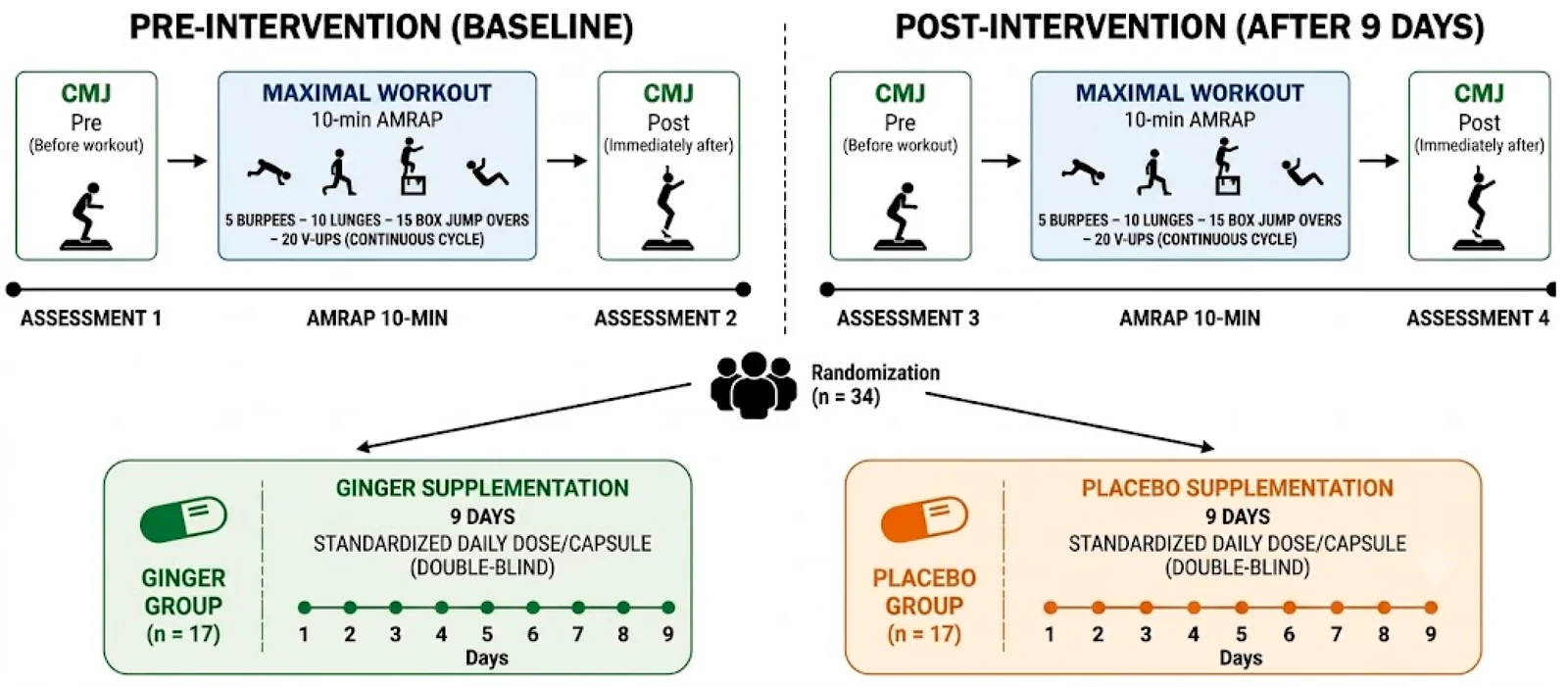
Schematic representation of the randomized double-blind intervention protocol. Created using Canva (https://www.canva.com/pro/; accessed on 30 May 2026).

This study employed a double-blind, placebo-controlled experimental design to investigate the effects of 9 days of ginger (*Zingiber officinale*) supplementation on performance and physiological responses in CrossFit^®^ athletes (Figure 1). The protocol consisted of four neuromuscular assessments using the countermovement jump (CMJ): before and immediately after a maximal CrossFit^®^ workout (10 min AMRAP), performed during two distinct periods (pre- and post-intervention). The study protocol was approved by the institutional ethics committee (Approval code: CAAE 35605220.1.0000.5147; Approval No. 4.366.750) and prospectively registered in the Brazilian Registry of Clinical Trials (REBEC RBR-8 × 3tmqc).

### 2.2. Participants

**Figure 2:**
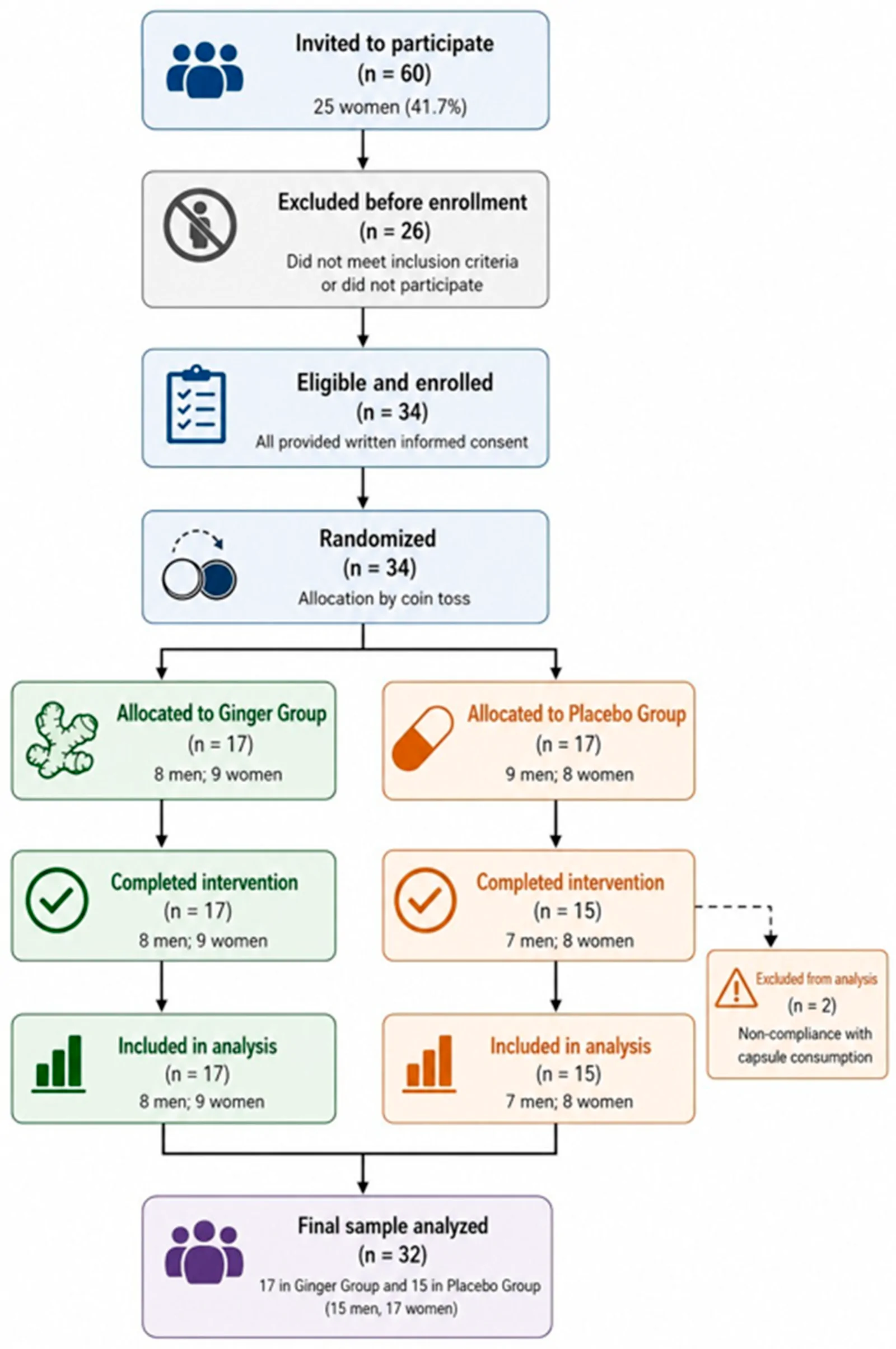
Flow diagram of participant recruitment, randomization, and inclusion in the final analysis. Created using Canva (https://www.canva.com/pro/; accessed on 30 May 2026).

A total of 60 individuals were invited to participate, of whom 25 were women (41.7%). Following recruitment and initial screening, 34 volunteers (56.7% of those invited) entered the study. All eligible participants provided written informed consent in accordance with the guidelines established by Resolution No. 466/12 of the Brazilian National Health Council. The minimum required sample size was estimated a priori using G*Power (version 3.1; Heinrich Heine University, Düsseldorf, Germany). Assuming a two-group comparison, a significance level of 5% (α = 0.05), statistical power of 80% (1 − β = 0.80), and a moderate effect size, the analysis indicated that a minimum of 30 participants (15 per group) would be required. After enrollment, participants were randomly allocated in a 1:1 ratio by coin toss to either the Ginger group (n = 17; 8 men and 9 women) or the Placebo group (n = 17; 9 men and 8 women). During data collection, two participants from the Placebo group (5.9%) were excluded from the final analysis due to non-compliance with the recommended capsule consumption. Consequently, the final sample consisted of 32 CrossFit^®^ practitioners (17 in the Ginger group and 15 in the Placebo group), exceeding the minimum sample size required by the a priori power analysis. A flow diagram summarizing participant recruitment, allocation, follow-up, and analysis is presented in Figure 2. All participants underwent anthropometric assessments and were instructed to maintain their usual physical activity habits, refrain from strenuous physical activity outside their regular CrossFit^®^ training routine, and avoid caffeine consumption during the 48 h preceding the experimental tests.

### 2.3. Anthropometric Assessment

Anthropometric measurements were obtained prior to the experimental procedures. Body mass was measured using a calibrated Filizola^®^ digital scale (Filizola S.A., São Paulo, Brazil), and height was assessed using a Seca^®^ wall-mounted stadiometer (Seca GmbH & Co. KG, Hamburg, Germany). Body mass index (BMI) was calculated as body mass (kg) divided by height squared (m^2^). Body density was estimated from skinfold thickness measurements using the sex-specific seven-site equations proposed by Jackson and Pollock [21] for men and by Jackson et al. [22] for women. Body fat percentage was subsequently estimated using the Brozek equation [22]. All skinfold measurements were obtained by the same trained evaluator using a Lange^®^ Skinfold Caliper (Beta Technology Inc., Cambridge, MD, USA) under standardized testing conditions. Participants were assessed wearing light clothing and no shoes.

### 2.4. AMRAP 10 Min Specific Performance Test and Fatigue Induction

**Figure 3:**
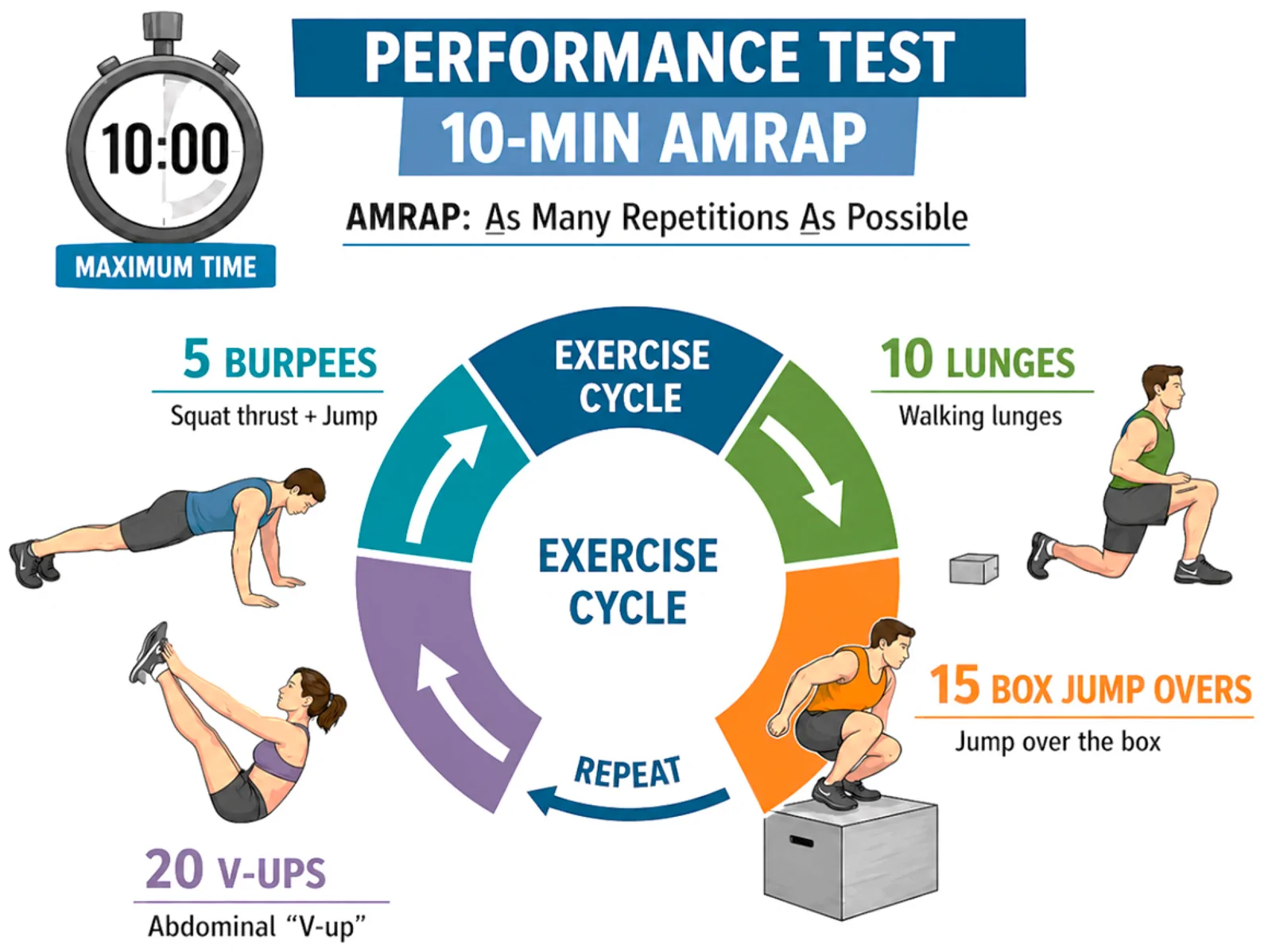
Representation of the AMRAP (As Many Repetitions As Possible) 10 min specific performance test. Created using Canva (https://www.canva.com/pro/; accessed on 30 May 2026).

Physical performance and metabolic capacity were assessed using a 10 min maximal protocol based on CrossFit^®^ competition formats, specifically designed to simulate high metabolic demands. The test followed the AMRAP (as many repetitions as possible) format, consisting of a continuous cycle of: 5 burpees, 10 lunges, 15 box jump overs, and 20 V-ups (totaling 50 repetitions per round). The final score was determined by the total number of repetitions completed. Beyond being a measure of specific performance, this protocol was utilized as a standardized fatigue induction task. The comparison between baseline and post-supplementation scores served as the primary indicator of the ergogenic effect of ginger on maximal work capacity. Perceptual responses were monitored throughout the test, with rating of perceived exertion (RPE) recorded immediately after each 10 min bout using the Borg 1–10 scale to evaluate the effects of supplementation on perceived fatigue [23]. To ensure data reproducibility and minimize potential sources of variability, all testing sessions were conducted at the same time of day under standardized environmental conditions by the same investigators. In addition, participants received standardized instructions and verbal encouragement throughout the AMRAP test. Figure 3 illustrates the sequence of the AMRAP test.

### 2.5. Neuromuscular Performance Assessment (CMJ) and Fatigue Index

Neuromuscular status was monitored through the Countermovement Jump (CMJ) performed on a force plate (PS-2142, PASCO^®^ Instrument Inc., Roseville, CA, USA). To isolate lower-body power and minimize the influence of the upper body, participants kept their hands on their hips throughout the movement. The CMJ protocol involved three trials performed at each time point with 60 s rest, and the best jump (highest height) was used for analysis. The study involved four distinct CMJ assessment points to track the neuromuscular response before and after the 9-day intervention: (a) CMJ 1: Baseline (Pre-fatigue, Pre-supplementation); (b) CMJ 2: Acute fatigue (Post-AMRAP 1, Pre-supplementation); (c) CMJ 3: Baseline (Pre-fatigue, Post-supplementation); (d) CMJ 4: Acute fatigue (Post-AMRAP 2, Post-supplementation).

The CMJ variables were selected based on their established sensitivity to neuromuscular fatigue and their ability to characterize explosive performance in athletic populations. Jump height, calculated using the impulse–momentum method, was included as a global indicator of vertical performance and lower-limb neuromuscular function [24]. Normalized peak power and peak force were analyzed as markers of explosive strength and maximal mechanical output [25]. To assess movement strategy and stretch-shortening cycle efficiency, the Reactive Strength Index-modified (RSI-mod) was calculated as the ratio of jump height to time to takeoff, a metric known to be highly sensitive to fatigue-induced alterations in jump mechanics [26]. In addition, relative braking rating of force development (RFD) and concentric impulse were examined to characterize force-time adaptations during the eccentric-to-concentric transition, variables that may detect acute neuromuscular fatigue more sensitively than jump height alone [27]. To quantify the magnitude of fatigue and examine the potential ergogenic effects of ginger supplementation, a Fatigue Index (%F) was calculated for each variable using the following equation: %F = [(Post-fatigue − Pre-fatigue)/Pre-fatigue] × 100.

### 2.6. Supplementation Protocol

Participants were randomly allocated to either the Ginger or Placebo group. Both groups followed a daily supplementation protocol for nine consecutive days, receiving a total dose of 2 g·day^−1^ administered through five capsules containing 400 mg each. Capsules provided to the Ginger group contained *Zingiber officinale* powder, whereas the Placebo group received capsules containing cornstarch. Capsules were identical in appearance, color, and odor, ensuring maintenance of the double-blind design. All compounds were prepared by a certified compounding pharmacy. Participants were instructed to maintain their habitual dietary patterns throughout the experimental period and to avoid intentional changes in energy intake or dietary composition. Additionally, they were instructed to refrain from consuming foods, beverages, or supplements containing ginger during the intervention period to prevent interference with the supplementation dose and to ensure standardized exposure to the bioactive compound. Participants were instructed to consume the entire daily dose once daily at approximately 7:00 a.m., together with breakfast and water, in order to minimize potential variations in absorption and gastrointestinal discomfort. Compliance with the supplementation protocol and dietary recommendations was monitored daily through direct WhatsApp^®^ messages. Participants were asked to confirm daily capsule consumption and report any deviations from the prescribed regimen. Participants who did not comply with the supplementation protocol were excluded from the final analyses.

### 2.7. Statistical Analysis

All data were initially tested for normality using the Shapiro–Wilk test and for homogeneity of variances using Levene’s test. Participant characteristics (age, body mass, height, BMI, and body fat percentage) were compared between the Ginger and Placebo groups using independent-samples t-tests. Statistical significance was set at *p* < 0.05 for all analyses. To evaluate the effects of supplementation on physical performance (AMRAP and RPE), a One-Way Analysis of Covariance (ANCOVA) was performed. In this model, the post-supplementation values were used as the dependent variables, the group (Ginger vs. Placebo) was the fixed factor, and the respective baseline (pre-supplementation) values were included as covariates to control for initial differences between participants. Similarly, to investigate the attenuation of neuromuscular fatigue, ANCOVAs were conducted for each CMJ variable using the Percentage of Fatigue (%F) as the dependent variable. Specifically, the post-supplementation %F (calculated from CMJ 4 vs. CMJ 3) was the dependent variable, while the pre-supplementation %F (calculated from CMJ 2 vs. CMJ 1) was used as the covariate. This approach allowed for a precise comparison of the fatigue response between groups while adjusting for each athlete’s individual baseline fatigue sensitivity. When significant interactions were detected, Bonferroni-adjusted post hoc tests were applied to identify specific differences between time points and groups. Effect sizes were reported as partial eta squared (ηp^2^), with values of 0.01, 0.06, and 0.14 interpreted as small, medium, and large effects, respectively [28]. All statistical analyses were performed using Python (version 3.x) with the Pandas, NumPy, and Statsmodels libraries.

## 3. Results

Descriptive statistics (mean ± SD and 95% confidence intervals) for all neuromuscular performance variables assessed at the four experimental time points (CMJ 1, CMJ 2, CMJ 3, and CMJ 4) in both the Ginger and Placebo groups are presented in Supplementary Table S1. No significant baseline differences were observed between the Ginger and Placebo groups for age, height, body mass, BMI, or body fat percentage (*p* > 0.05). Baseline characteristics of the participants are presented in Table 1.

**Figure 4:**
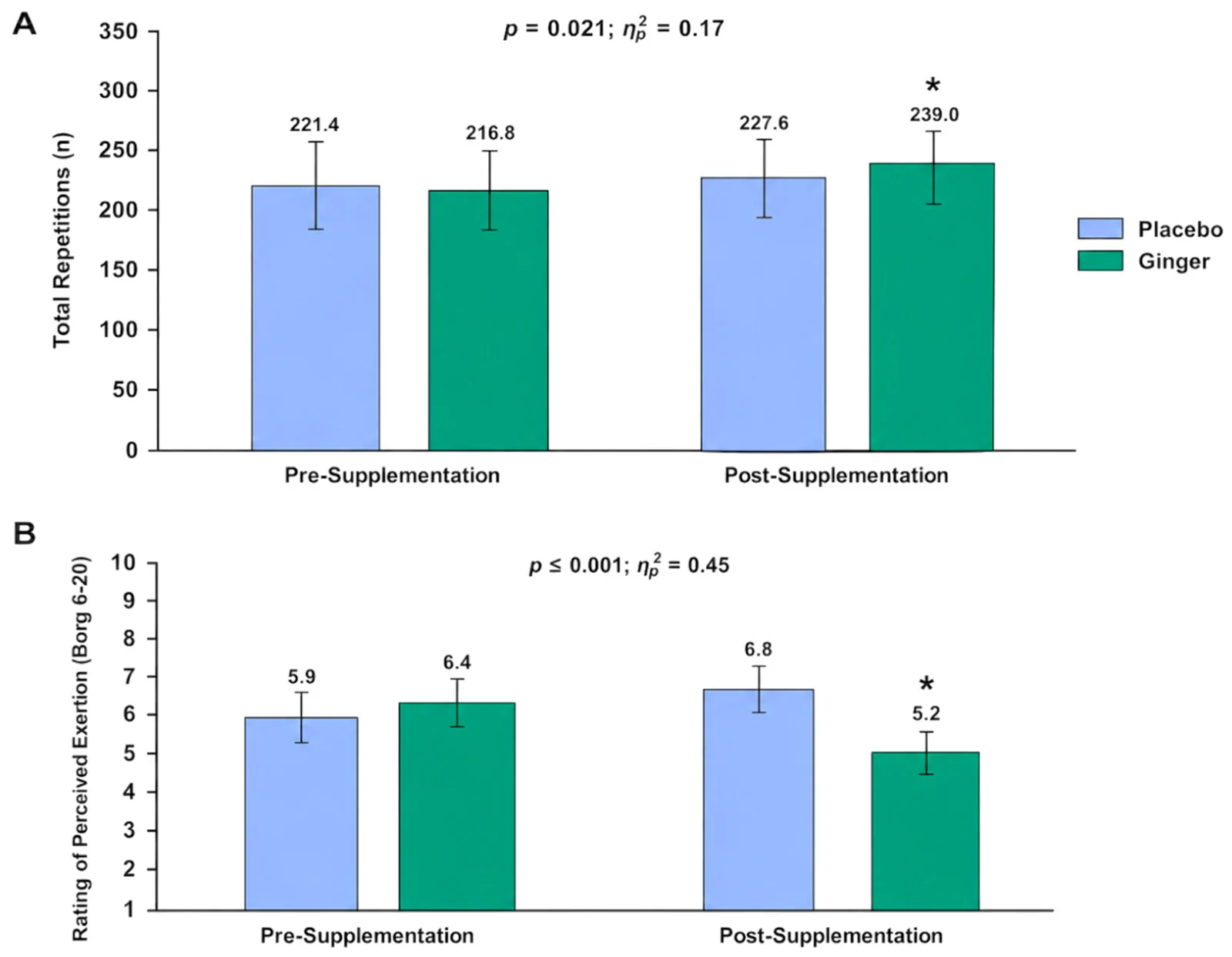
Data presented by means ± standard deviation for (**A**) total repetitions completed in the 10 min AMRAP, and (**B**) rating of perceived exertion measured before and after supplementation in the placebo and ginger groups. * *p* ≤ 0.021 vs. Placebo.

A significant effect of supplementation was observed for physical performance (F_1,29_ = 5.95; *p* = 0.021; ηp^2^ = 0.17), with the Ginger group demonstrating a significantly greater adjusted mean number of repetitions than the Placebo group (240.1 ± 29.8 vs. 227.5 ± 29.0 repetitions, respectively), corresponding to a moderate effect size (Figure 4). This improvement represented an absolute increase of 21.65 ± 19.04 repetitions (+10.47%) in the Ginger group, compared with only 4.80 ± 20.50 repetitions (+2.87%) in the Placebo group. Likewise, a significant effect of supplementation was observed for RPE (F_1,29_ = 24.11; *p* < 0.001; ηp^2^ = 0.45), with the Ginger group exhibiting a lower adjusted mean RPE than the Placebo group (5.2 ± 1.3 vs. 6.8 ± 1.0), corresponding to a large effect size. The Ginger group showed an absolute reduction in perceived exertion of 1.12 ± 0.78 arbitrary units (a.u.) (−17.45%), whereas the Placebo group exhibited an increase of 0.93 ± 1.79 arbitrary units (a.u.) (+28.28%).

The comparison of the neuromuscular fatigue index between groups revealed a significant difference only for peak force (F = 6.62, *p* = 0.015, ηp^2^ = 0.186). The Ginger group exhibited a positive fatigue index, whereas the Placebo group showed a reduction in peak force, indicating a lower degree of neuromuscular fatigue following supplementation (Table 2).

## 4. Discussion

This study aimed to investigate the effects of 9 days of ginger supplementation on physical performance and physiological responses in CrossFit^®^ athletes. Our primary findings demonstrate that ginger significantly enhanced maximal work capacity, as evidenced by improved performance in a 10 min AMRAP test. Furthermore, ginger significantly attenuated the RPE after the AMRAP, suggesting a reduced subjective load despite increased output. Crucially, the analysis of fatigue indices revealed that ginger supplementation significantly protected neuromuscular function, particularly in Peak Force, indicating enhanced resilience to acute exercise-induced fatigue. These results highlight the potential ergogenic benefits of ginger for CrossFit^®^ athletes, offering a natural strategy to improve performance and mitigate fatigue during high-intensity functional training [9,11].

Although the present study did not directly assess biochemical markers of inflammation, oxidative stress, or muscle damage, previous experimental evidence provides plausible mechanisms through which ginger may influence exercise performance and recovery. Therefore, the mechanistic explanations presented here should be interpreted as hypotheses supported by the existing literature rather than direct evidence from the present study. The improvements in AMRAP performance observed in the Ginger group are more likely to be related to the analgesic properties of its bioactive compounds, particularly 6-gingerol and 6-shogaol, than to an acute attenuation of exercise-induced inflammation. The marked reduction in perceived exertion suggests that ginger may have decreased nociceptor sensitization and modulated pain-related signaling pathways [10], enabling participants to tolerate higher levels of discomfort and sustain greater exercise intensity during the AMRAP. Although RPE is inherently subjective and may be influenced by individual perception, its standardized assessment under identical experimental conditions and its widespread use as a validated measure of internal exercise load support the interpretation of the present findings. Ginger also possesses well-established anti-inflammatory and antioxidant properties, although these mechanisms are more likely to contribute to post-exercise recovery and the preservation of neuromuscular function than to directly enhance performance during a single exercise bout. Experimental studies have shown that ginger can attenuate exercise-induced inflammation through inhibition of nuclear factor kappa-B, resulting in reduced cyclooxygenase-2 expression and lower prostaglandin E_2_ production [11,16,29,30]. In addition, emerging evidence suggests that 6-gingerol may enhance glycogen storage and strengthen endogenous antioxidant defenses, thereby reducing oxidative stress and supporting metabolic efficiency during exercise [13,30]. Collectively, these mechanisms may have contributed to the preservation of peak force observed in the present study and may increase resistance to fatigue during repeated high-intensity efforts, although they should be interpreted as plausible explanations rather than mechanisms directly demonstrated by our findings.

The effectiveness of ginger supplementation may also depend on the dose and duration of intake [16,19,29]. Previous randomized trials have demonstrated that daily doses of approximately 2 g for 5–11 days can reduce muscle soreness following eccentric exercise by nearly 23–25%, suggesting that relatively low doses are sufficient to attenuate exercise-induced pain [14,19]. Higher doses may provide additional benefits for recovery-related outcomes. For example, supplementation with 4 g·day^−1^ for five days before exercise accelerated the recovery of muscle strength after eccentric contractions, although no significant effects were observed on markers of muscle damage or overall soreness [16]. Similarly, recreational distance runners consuming approximately 1.4 g·day^−1^ for five days experienced reduced post-exercise soreness and a small improvement in vertical jump performance, despite no clear enhancement in running performance [13]. Furthermore, supplementation strategies combining ginger with other bioactive compounds, such as annatto, have been shown to reduce pain and help preserve lower-limb power following DOMS-inducing exercise [29]. Collectively, these findings suggest that previous evidence has been more consistent for recovery-related outcomes than for direct performance enhancement. Although higher doses may potentiate some effects on neuromuscular recovery, current evidence indicates that improvements in exercise performance, strength, or power remain modest and inconsistent across studies [16,30]. Therefore, the ergogenic potential of ginger appears to be influenced by dosage, supplementation duration, exercise modality, and the specific outcome being assessed, with the strongest evidence supporting its role in attenuating post-exercise soreness and facilitating recovery.

Despite the promising results observed, it is important to emphasize that ginger remains a relatively under-investigated compound within the context of high-intensity sports performance [9]. Our findings suggest that the ergogenic benefits of ginger may be primarily driven by its analgesic properties. The significant reduction in RPE and the increase in AMRAP repetitions indicate that athletes were able to tolerate higher levels of discomfort, likely due to the modulation of pain-related pathways by gingerols [12,31,32]. This attenuation of the subjective cost of exercise appears to be the most potent driver of the performance gains observed in our CrossFit^®^ cohort. On the other hand, the effects on CMJ variables as indicators of neuromuscular fatigue were relatively modest. While we observed significant protection in Peak Force, the overall impact on the neuromuscular system aligns with previous literature reporting small-to-moderate effects of ginger on physical performance [9,19,31]. This subtle response in the CMJ suggests that while ginger mitigates the perception of fatigue and preserves some mechanical output, it may not completely override the physiological fatigue induced by a maximal 10 min bout. These findings reinforce the notion that ginger is perhaps more effective as a recovery aid and a sensory modulator than as a direct stimulant of neuromuscular capacity.

The supplementation protocol was well tolerated by all participants. No adverse events or side effects related to ginger supplementation were reported during the 9-day intervention. This observation is consistent with previous studies indicating that short-term ginger supplementation at doses of approximately 2 g·day^−1^ is generally safe and well tolerated, with only occasional reports of mild gastrointestinal discomfort [10,14,16,17]. Nevertheless, future studies employing longer supplementation periods or higher doses should continue to monitor adverse events systematically to further establish the safety profile of ginger in athletic populations.

Despite the significant findings, this study has limitations that should be acknowledged. First, although our sample size met the a priori power requirements, it remained relatively small, which may have limited our ability to detect smaller differences in some CMJ variables that showed non-significant trends. Second, while the 9-day supplementation period was sufficient to observe ergogenic effects, longer-term interventions are needed to determine whether these benefits are sustained or reach a plateau over time. Third, we did not include direct biochemical markers of muscle damage (e.g., creatine kinase) or systemic inflammation (e.g., C-reactive protein and IL-6), which would have provided additional insight into the physiological mechanisms underlying the observed performance gains. Furthermore, the absence of a crossover design means that individual variability in response to ginger supplementation could not be fully controlled, although the use of ANCOVA and baseline-adjusted indices helped mitigate this limitation. Another limitation is that men and women were analyzed together. Although this approach was adopted to preserve statistical power, sex-related differences in physiological responses to exercise, fatigue, and nutritional interventions may have influenced the observed outcomes. Because the present study was not powered to detect sex-specific effects, future studies with larger samples should investigate whether the ergogenic effects of ginger differ between male and female CrossFit^®^ practitioners. Finally, adherence to the supplementation protocol was assessed through participant self-report rather than by objective methods such as capsule counts. Although compliance was monitored daily via WhatsApp^®^ and participants reporting non-compliance were excluded from the final analyses, future studies should incorporate objective measures of adherence to further strengthen methodological rigor. Future research should also investigate the dose–response relationship of standardized gingerol extracts in high-intensity functional training and explore their potential synergy with other recovery strategies. Additionally, longitudinal studies monitoring training volume and recovery over several weeks would help clarify the long-term impact of ginger supplementation on athletic development and injury prevention in CrossFit^®^ populations.

### Practical Application

The present findings suggest that short-term ginger supplementation may represent a practical nutritional strategy to improve performance and reduce perceived exertion during high-intensity functional exercise. The preservation of peak force also indicates a potential benefit for maintaining neuromuscular performance during demanding training sessions. However, because significant effects were observed in only one neuromuscular variable, ginger should be considered a complementary strategy rather than a substitute for established training, recovery, and nutritional practices.

## 5. Conclusions

In conclusion, nine days of ginger supplementation improved maximal physical performance in CrossFit^®^ athletes, as evidenced by an increased repetition count during a 10 min AMRAP test, and reduced ratings of perceived exertion. Ginger supplementation also attenuated the decline in peak force following exhaustive exercise, suggesting a potential benefit for preserving selected aspects of neuromuscular function. Although no significant effects were observed for the remaining neuromuscular variables, the present findings indicate that short-term ginger supplementation may represent a promising nutritional strategy to enhance performance during high-intensity functional exercise. Nevertheless, larger and longer-term studies are needed to confirm these findings and further clarify the ergogenic potential of ginger.

## Figures and Tables

**Table 1:** Comparison of baseline characteristics between the Placebo and Ginger groups.

Variable	Placebo	Ginger	Statistic	*p*-Value	Effect Size
Age (years)	32.3 ± 6.5 [28.7; 35.9]	35.8 ± 4.5 [33.5; 38.1]	t = 1.79	0.082	d’ = 0.63
Height (m)	1.7 ± 0.1 [1.65; 1.74]	1.7 ± 0.1 [1.67; 1.75]	t = −0.39	0.696	d’ = 0.14
Body Mass (kg)	75.4 ± 16.2 [66.5; 84.4]	75.2 ± 15.4 [67.3; 83.1]	t = 0.03	0.976	d’ = 0.01
BMI (kg·m^−2^)	25.9 ± 3.6 [24.0; 27.9]	25.6 ± 3.5 [23.8; 27.4]	t = 0.28	0.79	d’ = 0.1
Body Fat (%)	22.7 ± 10.2 [18.0; 29.0]	19.7 ± 7.0 [16.0; 23.0]	t = 1.19	0.349	d’ = 0.33

BMI—body mass index.

**Table 2:** Comparison of neuromuscular fatigue index between Placebo and Ginger groups.

Effect Size	*p*-Value	F-Value	Ginger (n = 17)	Placebo (n = 15)	Variable
0.090	0.101	2.87	−18.0 ± 16.4 [−26.4, −9.5]	−28.7 ± 16.9 [−38.0, −19.3]	Jump height (m)
0.117	0.059	3.86	5.0 ± 18.9 [−4.7, 14.8]	−6.0 ± 12.7 [−13.0, 1.0]	Peak power (N)
0.186	0.015	6.62	9.8 ± 23.8 [−2.4, 22.0] *	−10.3 ± 18.9 [−20.8, 0.1]	Peak force (N)
0.120	0.056	3.95	−12.6 ± 21.5 [−23.7, −1.6]	−26.9 ± 18.6 [−37.2, −16.6]	Center of mass displacement (m)
0.078	0.129	2.44	46.1 ± 132.9 [−22.3, 114.4]	−15.4 ± 63.8 [−50.7, 20.0]	Rating of force development braking (N/s/kg)
0.014	0.533	0.40	−17.1 ± 22.9 [−28.9, −5.3]	−24.9 ± 41.4 [−47.8, −1.9]	Reactive strength index modified
0.018	0.470	0.54	3.9 ± 34.5 [−13.8, 21.6]	−7.0 ± 27.8 [−22.4, 8.4]	Concentric impulse (N.s)

Note: Data are presented as Mean ± SD [95% Confidence Interval]. Neuromuscular fatigue index (%F) calculated as [(Post-fatigue − Pre-fatigue)/Pre-fatigue] × 100. *p*-values and effect sizes (ηp^2^) derived from ANCOVA using the pre-supplementation fatigue index as a covariate. * *p* = 0.015) vs. Placebo.

## Data Availability

The data associated with this study have been deposited in a publicly accessible Figshare repository (https://doi.org/10.6084/m9.figshare.32594976).

## Supplementary Materials

The following supporting information can be downloaded at: https://www.mdpi.com/article/10.3390/jfmk11030301/s1, Table S1: Descriptive data (Mean ± SD [95% CI]) for Countermovement Jump (CMJ) variables across four assessment moments.

## Abbreviations

The following abbreviations are used in this manuscript:

AMRAP	As Many Repetitions As Possible
ANCOVA	Analysis of Covariance
BMI	Body Mass Index
CAAE	Certificado de Apresentação para Apreciação Ética
CAPES	Coordenação de Aperfeiçoamento de Pessoal de Nível Superior
CMJ	Countermovement Jump
RFD	Rating of Force Development
RPE	Rating of Perceived Exertion
ReBEC	Brazilian Registry of Clinical Trials
SD	Standard Deviation
%F	Fatigue Index

## References

[1] Aravena-Sagardia P., Barramuño-Medina M., Vásquez B.P., Pichinao Pichinao S., Sepúlveda P.R., Herrera-Valenzuela T., Hernandez-Martinez J., Levín-Catrilao Á., Villagrán-Silva F., Vásquez-Carrasco E. (2025). Effects of a CrossFit Training Program on Body Composition and Physical Fitness in Novice and Advanced Practitioners: An Inter-Individual Analysis. Appl. Sci..

[2] Ponce-García T., García-Romero J., Carrasco-Fernández L., Castillo-Domínguez A., Benítez-Porres J. (2025). Sex differences in anaerobic performance in CrossFit^®^ athletes: A comparison of three different all-out tests. PeerJ.

[3] Rios M., Cardoso R., Reis V.M., Moreira-Gonçalves D., Pyne D.B., Fernandes R.J. (2025). Sex-related differences in the acute physiological response to a high-intensity CrossFit^®^ workout. Curr. Res. Physiol..

[4] Sauvé B., Haugan M., Paulsen G. (2024). Physical and physiological characteristics of elite CrossFit athletes. Sports.

[5] Jowsey J.R., Haff G.G., Comfort P., Ripley N.J. (2025). Performance in Multi-Joint Force-Plate Assessments in Male and Female CrossFit^®^ Athletes. Biomechanics.

[6] Maté-Muñoz J.L., Lougedo J.H., Barba M., Cañuelo-Márquez A.M., Guodemar-Pérez J., García-Fernández P., Lozano-Estevan M.D.C., Alonso-Melero R., Sánchez-Calabuig M.A., Ruíz-López M. (2018). Cardiometabolic and Muscular Fatigue Responses to Different CrossFit^®^ Workouts. J. Sports Sci. Med..

[7] Martinho D.V., Rebelo A., Clemente F.M., Costa R., Gouveia É.R., Field A., Casonatto J., van den Hoek D., Durkalec-Michalski K., Ormsbee M.J. (2025). Nutrition in CrossFit^®^–scientific evidence and practical perspectives: A systematic scoping review. J. Int. Soc. Sports Nutr..

[8] dos Santos Quaresma M.V.L., Marques C.G., Nakamoto F.P. (2021). Effects of diet interventions, dietary supplements, and performance-enhancing substances on the performance of CrossFit-trained individuals: A systematic review of clinical studies. Nutrition.

[9] Kurtz J., Watson M., Robinson B., Edmondson C., Wentz L. (2026). The Influence of Ginger Supplementation on Cycling Performance. Sports.

[10] Broeckel J., Estes L., Leonard M., Dickerson B., Gonzalez D., Purpura M., Jäger R., Sowinski R., Rasmussen C., Kreider R. (2025). Effects of ginger supplementation in individuals with mild to moderate joint pain I: Ratings of pain, functional capacity, and markers of inflammation. J. Int. Soc. Sports Nutr..

[11] Karizak S., Shahnehzad M., Zar A. (2024). Impact of ginger supplementation on serum PGE2, COX2, and IL-6 in response to exhaustive exercise in female taekwondo athletes. Comp. Exerc. Physiol..

[12] Tafrishinejad A., Farsani H., Heidari-Soureshjani S., Sherwin C., Azadegan-Dehkordi Z. (2023). The analgesic effect of ginger on postoperative pain: A systematic review of clinical trials. Nat. Prod. J..

[13] Wilson P.B. (2020). A randomized double-blind trial of ginger root for reducing muscle soreness and improving physical performance recovery among experienced recreational distance runners. J. Diet. Suppl..

[14] Black C., Herring M., Hurley D., O’Connor P. (2010). Ginger (Zingiber officinale) reduces muscle pain caused by eccentric exercise. J. Pain Off. J. Am. Pain Soc..

[15] Anugrah S.M., Kusnanik N.W., Wahjuni E.S., Muhammad H.N., Sulistyarto S., Purwanto B., Resmana D., Juniarsyah A.D., Ayubi N., Sari E. (2024). Herbal supplements that have the potential to accelerate recovery of exercise-induced muscle damage: Systematic review. Retos.

[16] Matsumura M., Zavorsky G.S., Smoliga J. (2015). The Effects of Pre-Exercise Ginger Supplementation on Muscle Damage and Delayed Onset Muscle Soreness. Phytother. Res..

[17] Wilson P.B., Fitzgerald J.S., Rhodes G.S., Lundstrom C.J., Ingraham S.J. (2015). Effectiveness of ginger root (*Zingiber officinale*) on running-induced muscle soreness and function: A pilot study. Int. J. Athl. Ther. Train..

[18] Wilson P.B. (2015). Ginger (*Zingiber officinale*) as an analgesic and ergogenic aid in sport: A systemic review. J. Strength Cond. Res..

[19] Black C., O’Connor P. (2010). Acute effects of dietary ginger on muscle pain induced by eccentric exercise. Phytother. Res..

[20] Barba-Ruíz M., Hermosilla-Perona F., Heredia-Elvar J.R., Gómez-González N., Da Silva-Grigoletto M.E., Muriarte-Solana D. (2024). Muscular performance analysis in “cross” modalities: Comparison between “AMRAP,” “EMOM” and “RFT” configurations. Front. Physiol..

[21] Jackson A.S., Pollock M.L. (1978). Generalized equations for predicting body density of men. Br. J. Nutr..

[22] Jackson A.S., Pollock M.L., Ward A. (1980). Generalized equations for predicting body density of women. Med. Sci. Sports Exerc..

[23] Zourdos M.C., Klemp A., Dolan C., Quiles J.M., Schau K.A., Jo E., Helms E., Esgro B., Duncan S., Merino S.G. (2016). Novel resistance training–specific rating of perceived exertion scale measuring repetitions in reserve. J. Strength Cond. Res..

[24] Claudino J.G., Cronin J., Mezêncio B., McMaster D.T., McGuigan M., Tricoli V., Amadio A.C., Serrão J.C. (2017). The countermovement jump to monitor neuromuscular status: A meta-analysis. J. Sci. Med. Sport.

[25] Gathercole R., Sporer B., Stellingwerff T., Sleivert G. (2015). Alternative countermovement-jump analysis to quantify acute neuromuscular fatigue. Int. J. Sports Physiol. Perform..

[26] Suchomel T.J., Bailey C.A., Sole C.J., Grazer J.L., Beckham G.K. (2015). Using reactive strength index-modified as an explosive performance measurement tool in Division I athletes. J. Strength Cond. Res..

[27] Wu P.P.-Y., Sterkenburg N., Everett K., Chapman D.W., White N., Mengersen K. (2019). Predicting fatigue using countermovement jump force-time signatures: PCA can distinguish neuromuscular versus metabolic fatigue. PLoS ONE.

[28] Yagin F.H., Pinar A., de Sousa Fernandes M.S. (2024). Statistical effect sizes in sports science. J. Exerc. Sci. Phys. Act. Rev..

[29] Domínguez-Balmaseda D., Diez-Vega I., Larrosa M., Juan A.S., Issaly N., Moreno-Pérez D., Burgos S., Sillero-Quintana M., González C., Bas A. (2020). Effect of a Blend of Zingiber officinale Roscoe and Bixa orellana L. Herbal Supplement on the Recovery of Delayed-Onset Muscle Soreness Induced by Unaccustomed Eccentric Resistance Training: A Randomized, Triple-Blind, Placebo-Controlled Trial. Front. Physiol..

[30] Rondanelli M., Fossari F., Vecchio V., Gasparri C., Peroni G., Spadaccini D., Riva A., Petrangolini G., Iannello G., Nichetti M. (2020). Clinical trials on pain lowering effect of ginger: A narrative review. Phytother. Res..

[31] Demirli A., Ulupınar S., Terzi M., Özbay S., Özkara A.B., Gençoğlu C., Ouergui I., Ardigò L. (2025). Synergistic Effects of Green Tea Extract and Ginger Supplementation on Endurance Performance and Thermal Perception in Normothermic and Cold Environments: A Randomized, Placebo-Controlled, Double-Blind Crossover Trial. Nutrients.

[32] Kim S., Cheon C., Kim B., Kim W. (2022). The Effect of Ginger and Its Sub-Components on Pain. Plants.

