# Supplementary material for: Ginger Supplementation Improves Performance and Attenuates Perceived Exertion and Neuromuscular Fatigue in CrossFit® Athletes
Source: J Funct Morphol Kinesiol. 2026 Jul 29;11(3):301. doi: 10.3390/jfmk11030301 (PMC13510715; doi:10.3390/jfmk11030301)
Supplement: Supplementary file 1 [file jfmk-11-00301-s001.zip › jfmk-4393149-supplementary.pdf]

Supplementary Table S1. Descriptive data (Mean ± SD [95% CI]) for Countermovement Jump (CMJ) variables across four assessment moments.

Variable	Group	CMJ 1	CMJ 2	CMJ 3	CMJ 4
Jump Height (m)	Ginger	0.305 ± 0.078 [0.265; 0.346]	0.28 ± 0.068 [0.245; 0.314]	0.355 ± 0.083 [0.313; 0.398]	0.286 ± 0.071 [0.250; 0.323]
	Placebo	0.295 ± 0.042 [0.272; 0.319]	0.263 ± 0.074 [0.222; 0.304]	0.373 ± 0.121 [0.306; 0.44]	0.25 ± 0.046 [0.224; 0.276]
Reactive Strength Index Modified	Ginger	0.388 ± 0.132 [0.32; 0.456]	0.343 ± 0.107 [0.288; 0.399]	0.456 ± 0.102 [0.404; 0.508]	0.38 ± 0.144 [0.306; 0.453]
	Placebo	0.384 ± 0.074 [0.344; 0.425]	0.335 ± 0.072 [0.296; 0.375]	0.440 ± 0.102 [0.384; 0.497]	0.307 ± 0.131 [0.235; 0.38]
Center of Mass Displacement (m)	Ginger	0.307 ± 0.065 [0.274; 0.341]	0.275 ± 0.062 [0.243; 0.307]	0.344 ± 0.084 [0.301; 0.387]	0.293 ± 0.075 [0.255; 0.331]
	Placebo	0.314 ± 0.055 [0.283; 0.344]	0.28 ± 0.084 [0.233; 0.326]	0.346 ± 0.115 [0.283; 0.410]	0.237 ± 0.044 [0.213; 0.262]
Peak Force (N)	Ginger	1,810.7 ± 443.9 [1,582.4; 2,038.9]	1,472.9 ± 355.0 [1,290.4; 1,655.4]	1,374.8 ± 313.9 [1,213.4; 1,536.2]	1,457.6 ± 220.9 [1,344.0; 1,571.1]
	Placebo	1,943.7 ± 294.0 [1,780.9; 2,106.5]	1,609.3 ± 274.8 [1,457.1; 1,761.5]	1,446.1 ± 285.9 [1,287.8; 1,604.4]	1,268.1 ± 233.0 [1,139.0; 1,397.1]
Peak Power (W/kg)	Ginger	46.6 ± 9.1 [42.0; 51.3]	38.6 ± 8.6 [34.2; 43.1]	46.5 ± 10.8 [41.0; 52.1]	47.8 ± 9.1 [43.1; 52.5]
	Placebo	44.5 ± 6.1 [41.1; 47.9]	35.1 ± 4.9 [32.4; 37.8]	46.6 ± 6.0 [43.3; 49.9]	43.2 ± 3.3 [41.4; 45.0]
RFD Braking (N/s/kg)	Ginger	92.3 ± 48.8 [67.2; 117.4]	92.3 ± 45.8 [68.7; 115.9]	97.5 ± 58.4 [67.5; 127.5]	108.5 ± 62.0 [76.6; 140.4]
	Placebo	102.0 ± 66.0 [65.5; 138.5]	114.9 ± 59.6 [82.0; 147.9]	105.2 ± 61.6 [71.1; 139.3]	77.3 ± 52.8 [48.0; 106.5]
Concentric Impulse (N.s)	Ginger	151.4 ± 45.0 [128.3; 174.5]	152.5 ± 51.7 [125.9; 179.1]	134.8 ± 40.2 [114.1; 155.4]	132.1 ± 29.3 [117.0; 147.2]
	Placebo	168.2 ± 33.1 [149.9; 186.5]	156.9 ± 30.4 [140.1; 173.8]	149.8 ± 37.9 [128.8; 170.8]	136.2 ± 45.6 [110.9; 161.5]

Note: CMJ 1 = Baseline; CMJ 2 = Post-AMRAP 1 (Pre-supplementation); CMJ 3 = Post-9d (Pre-fatigue); CMJ 4 = Post-AMRAP 2 (Post-supplementation). CI = Confidence Interval.
